# Action Unit Detection by Learning the Deformation Coefficients of a 3D Morphable Model

**DOI:** 10.3390/s21020589

**Published:** 2021-01-15

**Authors:** Luigi Ariano, Claudio Ferrari, Stefano Berretti, Alberto Del Bimbo

**Affiliations:** Media Integration and Communication Center, University of Florence, 50134 Firenze, Italy; luigi.ariano@stud.unifi.it (L.A.); claudio.ferrari@unifi.it (C.F.); stefano.berretti@unifi.it (S.B.)

**Keywords:** 3D Morphable Model, dictionary learning, 3DMM deformation coefficients, Action Unit detection, Action Unit synthesis

## Abstract

Facial Action Units (AUs) correspond to the deformation/contraction of individual facial muscles or their combinations. As such, each AU affects just a small portion of the face, with deformations that are asymmetric in many cases. Generating and analyzing AUs in 3D is particularly relevant for the potential applications it can enable. In this paper, we propose a solution for 3D AU detection and synthesis by developing on a newly defined 3D Morphable Model (3DMM) of the face. Differently from most of the 3DMMs existing in the literature, which mainly model global variations of the face and show limitations in adapting to local and asymmetric deformations, the proposed solution is specifically devised to cope with such difficult morphings. During a training phase, the deformation coefficients are learned that enable the 3DMM to deform to 3D target scans showing neutral and facial expression of the same individual, thus decoupling expression from identity deformations. Then, such deformation coefficients are used, on the one hand, to train an AU classifier, on the other, they can be applied to a 3D neutral scan to generate AU deformations in a subject-independent manner. The proposed approach for AU detection is validated on the Bosphorus dataset, reporting competitive results with respect to the state-of-the-art, even in a challenging cross-dataset setting. We further show the learned coefficients are general enough to synthesize realistic 3D face instances with AUs activation.

## 1. Introduction

The automatic generation and recognition of the face dynamics that allow people to perform facial expressions has a strong theoretical and practical interest in a broad range of disciplines. It is well recognized that facial expressions as well as more subtle facial movements represent important visual cues that can support machine-based approaches aimed at the analysis of the human emotional state. In turn, this has a lot of potential applications such as monitoring for fatigue detection, gaming, deception recognition, or Human Computer Interaction, to cite a few.

From a physiological point of view, facial expressions can be seen as resulting from the contraction of facial muscles that deform the visible skin tissue. Usually, the type and intensity of facial expressions depend in a complex way on the level of activation of several muscles and their combined effect. Looking to the deformation of individual muscles or combination of them, Ekman and Friesen defined the Facial Action Coding System (FACS) [1] that relates facial expressions with the activation of Action Units (AUs), i.e., deformations of individual muscles. Both expression recognition and AU detection have been addressed in the literature using either RGB images or 3D data. The most common solution is that of recognizing and classifying the facial movements by analyzing the variation between neutral and expressive faces of some face features. In case of RGB imagery, these are extracted from the texture and are meant to describe appearance changes as induced by the facial deformations. In 3D, they instead capture changes occurring in the geometrical structure of the face surface. In both the cases, it is possible to use those features to discriminate between deformations that induced specific appearance changes. In other words, given a sample face, either in the form of RGB image or 3D data, we can observe the sample and decide which process, i.e., muscular movement, generated the particular appearance. However, operating this way we do not gather knowledge on the generative process itself, and so cannot replicate it. A way of addressing this problem would be that of directly analyzing the motions induced by AUs rather than what changes they determine on the face. Clearly, using 3D data is necessary to this aim, and would allow a direct evaluation of geometric deformations in the 3D space where they actually take place, rather than from their 2D projection in RGB images [2].

In this paper, we address the problem from the latter point of view, and we propose a method for AU detection in 3D using a 3D Morphable Model (3DMM) of the face. Given the local nature of action units, we developed on a particular variant of the 3DMM, called Sparse and Locally Coherent (SLC) 3DMM [3], which has the capability of modeling local deformations of the face. The SLC-3DMM can reproduce both structural face deformations related to the identity, and facial expressions. In fact, differently from most existing 3DMMs, its training data include neutral as well as expressive scans, which enables the 3DMM to reproduce also expression-like deformations. In addition, thanks to its particular learning formulation, the deformation components derived by the SLC solution capture quite well local deformations of the face. All of the above make it particularly suitable for our purposes.

This 3DMM can be efficiently fit to a target 3D scan using an iterative process that results into a 3D reconstructed model of the target subject, which is semantically consistent, i.e., in dense correspondence, with the 3DMM. In doing so, a set of deformation coefficients (weights) balancing the contribution of each atom are identified. In the most general case, such coefficients encode both structural deformations of the shape, commonly related to the identity of the subject, and other deformations that are instead related to expressions. In our case, we are interested in extrapolating the information related to the facial motions caused by the activation of AUs. In order to decouple the two, the proposed approach first adapts an average face to a 3D scan of an arbitrary individual in neutral expression. In this way, we account for the identity component. Then, the fitted neutral scan is used in place of the generic face to fit an expressive 3D face scan of the same subject. This two-step process allows us to disentangle the identity traits and the ones related to AUs activation. Once all these coefficients are collected from a training set of subjects, we look for recurrent patterns that identify facial motions related to AUs activation, and we use them to train a SVM and perform AU detection. In addition, with the proposed solution, we can easily recover AU-specific coefficients that can be used to deform a generic 3D model in neutral expression and synthesize a corresponding model with AU activation. This is achieved by simply averaging over all the collected coefficients that correspond to a particular AU, which demonstrates the effectiveness of the proposed two-step fitting solution.

A conceptually similar solution to learn expression-specific 3DMM coefficients and synthesize expressive face images appeared in [4]. There are yet several differences with respect to [4], the main one being that the above method dealt with 2D images rather than 3D faces. The 3DMM fitting was based on a set of sparse landmarks, and it aimed at modeling prototypical macro-expressions such as happiness or anger using the global DL-3DMM of [5]. These two characteristics prevented the method in [4] to account for smaller face motions. We instead perform here a way finer task that is modeling, classifying, and reproducing each muscular movement separately and directly in the 3D space. A set of sparse landmarks would result ineffective to this aim; being designed for coarsely localizing a sparse set of key-points on the face, they would not provide sufficiently detailed information. For this reason, the solution we present here uses a 3DMM, which is capable of accounting for such finer deformations using a dense 3D-3D fitting approach.

In summary, the novel contributions of this work are as follows:

- We propose a framework for learning the 3DMM deformation coefficients that control fine-grained, AU-specific deformations;
- We successfully apply them to the task of 3D AU detection in a challenging cross-database scenario, and we report competitive performance with respect to methods relying on carefully designed surface descriptors;
- We demonstrate the effectiveness and versatility of this solution by synthesizing realistic 3D faces with AUs activation using the learned coefficients.

The remainder of the paper is organized as follows: In Section 2, we revise and discuss works in the literature that are most closely related to our proposal. In Section 3, we summarize the characteristics of the SLC-3DMM used in this work, and we describe the method employed to perform the 3D-3D fitting. In Section 4, we present the proposed solution to learn AU-specific deformation coefficients from a set of raw 3D scans. These coefficients are then used to detect AUs and also to synthesize AUs from a generic 3D face model. Both a quantitative and a qualitative evaluation of AUs detection and synthesis are reported in Section 5. Finally, discussion and conclusions are given in Section 6 and Section 7.

## 2. Related Work

Our proposed approach is based on the idea of using a 3DMM of the face to detect AUs in 3D. So, works in the literature that propose solutions for 3DMM construction and 3D AU detection are relevant to our proposal. In the following, we summarize methods that focus on these two research topics in Section 2.1 and Section 2.2, respectively.

### 2.1. 3D Morphable Face Models

Our intent here is to evidence which are the main solutions appearing in the literature for constructing a statistical 3D model of the face, the main factors that characterize and differentiate them, and how these impact on our particular application.

First, we observe that two aspects are decisive in characterizing the different methods for 3DMM construction: (i) the variability in the human face, which is captured by the scans used as training set for the 3DMM. This variability directly depends on the number of scans and their heterogeneity; (ii) the method used for learning the deformation components that can modify the average model into a target one. The relevance of the first point is related to the fact the standard 3DMM cannot replicate deformations that do not appear in the training data, like facial expressions [5]. The second point, instead, determines the extent to which the 3DMM can capture the latent structure contained in the training data and use this to generalize to unseen samples.

The first work presenting a complete framework for 3DMM training and fitting was presented by Blanz and Vetter [6]. In their contribution, a 3DMM was derived independently for face shape and texture by using Principal Component Analysis (PCA) to identify the principal direction of the vector space spanning the training set of 3D scans. In this view, each 3D face scan is interpreted as a training sample, and shape and texture are transformed into a vector space representation. This seminal work largely influenced most of the subsequent literature on 3DMMs. In particular, the Basel Face Model (BFM) by Paysan et al. [7] refined the original 3DMM proposal into a publicly available tool. However, both the original 3DMM formulation and the BFM did not include expressive scans in the training data. Cao et al. [8] proposed a popular multi-linear 3D face model, called FaceWarehouse (FWH), that introduced expressive scans in the 3DMM training set. The idea here is to construct two separate linear models: one for the identities, using neutral scans, and one for expressions that are then linearly combined together. The method by Li et al. [9] improved the FaceWarehouse model by proposing FLAME, a powerful multi-linear PCA model composed of shape, expression blendshapes, and pose parameters that are learned separately from 4D sequences. Compared to FaceWarehouse, this model uses a larger number of training scans, demonstrating the advantage of using more abundant data in the training set. The methods above were all based on PCA, which provides a global decomposition of the face, i.e., modifying the average morphable model by acting on an individual deformation component has an effect on most of the points of the 3D face.

Deviating from this mainstream research, some methods investigated other ways than PCA to learn the deformation components. For example, Brunton et al. [10] used wavelet decomposition to define a multi-linear model, and they showed the advantage of learning localized and de-correlated components to deal with identity and expression variations in a 3D-3D fitting scenario. They also showed that by selecting proper masks of the face, it is possible to achieve higher robustness to occlusions with respect to a global model. Limitations are given by the fact that fitting the model to expressive faces requires an initialization based on facial landmarks as well as a dedicated, non-standard deformation procedure. The work by Lüthi et al. [11] also exposed the capability of modeling local and spatially uncorrelated deformations. This was obtained by generalizing the PCA-based statistical shape model using a Gaussian Process 3DMM. Authors of this work also elaborated on the importance of de-correlating facial movements to achieve more flexibility, but the local deformations learned by their model were not able to fully respect the anatomical structure of the face. As a consequence, it was difficult to directly apply such components for generating realistic face instances. A sparse variant of PCA with additional local support constraints to achieve localized yet realistic deformations was proposed by Neumann et al. [12]. However, in this work deformation components were learned on mesh sequences of single subjects, and they were used mainly for artistic and animation purposes. In the work by Ferrari et al. [5], a dictionary learning approach was used instead of PCA to derive the deformation components of the 3DMM. With this solution, called Dictionary Learning-based 3DMM (DL-3DMM), the deformation components combine together shape and expression variations into a single model. This increases the modeling capabilities by removing the orthogonality constraint as imposed by PCA, but components still cannot perform sparse local deformations.

Recently, solutions that apply Deep Neural Networks (DNNs) to learn non-linear 3D face models have been proposed. The idea presented in these methods is that of regressing shape and texture parameters directly from a face image [13], or a UV map [14]. Some of these solutions also show the capability of modeling extreme expressions using convolutional mesh autoencoders [15,16]. For example, Liu et al. [17] learned a non-linear face model from a huge set of raw 3D scans. Point clouds of the face are converted into identity and expression latent representations using the PointNet architecture [18], which results in a global shape model. A dense point-to-point correspondence among point sets is also established. This requires the training data to also include synthetic scans for which a dense correspondence is known, so resembling a semi-supervised setup. This method is also capable of dealing with scans from different databases.

In this work, we use the 3DMM defined by Ferrari et al. [3]. The model is learned from scans of the BU-3DFE database that includes both neutral and expressive faces with varying intensity, from low to exaggerated. The distinguishing trait of this 3DMM solution with respect to the existing literature is that vertices of the training scans are considered as independent samples. So, the number of training samples is fixed, while the number of training scans defines the dimensionality of each sample. This shows to be advantageous to extrapolate more patterns from the data, increasing the modeling capabilities. Operating this way, the resulting model also reduces the impact of the correlation between regions of the same face, allowing the learning of local deformations associated to both identity and expression in a single model, while ensuring realistic and interpretable deformations.

### 2.2. Action Unit Detection in 3D

Facial expression recognition and AU detection have been investigated primarily using 2D data [19,20], and there are not many works addressing AU detection from 3D scans. Despite AUs are activated by facial muscles, which implies a deformation of the 3D face surface, their definition and detection are based on the appearance changes that such movements induce on the texture. For example, when raising the eyebrows, wrinkles appear in the forehead; these kind of visual clues are normally exploited to perform AUs detection on 2D images. In 3D, instead, face analysis has been performed either using feature-based or model-based solutions. In the following, we summarize methods in these two broad categories.

Feature-based methods extract geometric descriptors from 3D facial scans either at a global or local level. One of the first works proposing AU detection in 3D was presented by Sandbach et al. [21]. They proposed to capture surface information by computing the Local Normal Binary Patterns (LNBPs) descriptors. They proposed two variants of the LNBPs: in the first variant, called LNBP${}_{OA}$, the scalar product between the normal of a central point and a point in the neighborhood is computed; in the second variant, called LNBP${}_{TA}$, the difference in the azimuth and elevation angles of two normals is evaluated. The LNBPs capture some shape information of the face and so of the corresponding action unit that deforms the face. After this feature extraction step, a Gentle-Boost algorithm is used for selecting the most relevant features. Action unit detection was finally obtained by training a SVM for each action unit. Authors also tested a different shape descriptor, i.e., the 3D-LBP descriptor [22] that applies the LBP operator to the depth map of the face. Feature fusion between 3D-LBP and each of the LNBP descriptors was applied as final step. The advent of Convolutional Neural Networks (CNN) has recently changed the way features are learned and classified also for the task of AU detection. For example, Li et al. [23] encoded texture and geometric information separately, by dedicating a sub-network to each descriptor. Geometric attributes were computed on the face mesh, then geometry maps were derived by displaying them as 2D images. A subsequent feature-level fusion network was used to combine together shape and texture descriptors. In the work by Taha et al. [24], data level fusion of texture and geometric information was used. This was obtained by first mapping the geometric descriptors onto texture images, so that three-channel images can be rendered and used as CNN input. However, these are both hybrid solutions that combine together texture and shape information. In addition, the 3D information is not used as is, but rather transformed to the 2D format in order to be processed by standard CNN architectures. CNNs solutions also lack of explainability, which is instead very important for face applications.

The other main category of methods we consider in our literature summary is that including model-based approaches. These methods exploit some prior information, which is typically in the form of a 3DMM of the face. In this context, the 3DMM is used to derive a dense point-to-point correspondence between the 3DMM itself and a target scan. This accounts both for rigid and non-rigid transformations. The representation of the target model, which is used for classification is then given by the deformation parameters that transform the 3DMM to the target scan. One of the first work following this paradigm was proposed by Mpiperis et al. [25] who used a bi-linear deformable model to characterize non-rigid facial deformations. In the work of Gong et al. [26], the shape of a 3D scan where the imaged subject shows a facial expressions was decomposed into a neutral part and an expressive part. The expressive part was then used for encoding the shape information to be used for classification. Tie et al. [27] focused on defining a generic model-based paradigm for recognizing emotions. In this work, the 3DMM was applied to video sequences with the aim of tracking and detecting facial expressions. Classification was obtained with a D-Isomap-based classifier.

We conclude by mentioning some works that developed on the idea of combining together the strengths of feature-based and model-based techniques. This idea was followed in the work by Zhen et al. [28], where the movement of facial muscles was used to segment the face into several regions. These regions were then used to compute geometric descriptors, and the decisions from different regions were fused using a weighted fusion scheme. An approach for detecting action units from 3D facial landmarks was recently proposed by Hinduja and Canavan [29]. To this end, they tracked the 3D landmarks using index-based statistical shape model, and trained a 2D CNN on 3D facial landmarks. They tried both binary and multi-class action unit classification providing experiments on 3D dynamic datasets (4D).

Looking to the above works, it is quite evident the lack of solutions that use a 3DMM in a fully 3D scenario, capable of extracting meaningful 3DMM deformation components for both AU detection and AU generation. Indeed, extracting components for both such tasks would be a clear confirmation of their capability of explaining local deformations of the face.

## 3. 3D Morphable Model

In the literature on 3DMM construction, there are some aspects that emerge quite evidently and that have a relevant impact on the modeling capabilities of the resulting tool. The first aspect that is worth mentioning is related to the set of scans used for learning the model. It is quite evident that the variability of the human face captured by the 3DMM is a direct consequence of the number of scans included in the training data and their heterogeneity. The second observation descends from the fact facial expressions and action units correspond to local deformations of the face due to the contraction of individual muscles or combinations of few of them. So, in order to include such local deformations among those that can be modeled, it is very important that scans with variegated local deformations of the face are observed in the training.

The two characteristics mentioned above are not commonly exposed jointly by the 3DMM existing in the literature. To the best of our knowledge, the Sparse and Locally-Coherent (SLC)-3DMM proposed by Ferrari et al. [3] shows the unique characteristic of having a large spectrum of variability in the generated models, including gender, ethnicity and expression, combined with the capability of modeling local deformations of the face. Based on this, we propose to use the SLC-3DMM for AUs detection from 3D scans. To this end, in the remaining parts of this section, we present the main solutions for constructing the model (Section 3.1) and fitting it in a dense way to a target scan (Section 3.2). These solutions were originally presented in [3], and are summarized here with the aim to make the paper as self-contained as possible. A full description of the SLC-3DMM method with all the details can be found in [3].

### 3.1. SLC-3DMM Construction

As we have mentioned above, the set of scans used to train the 3DMM is of great importance for determining its final modeling and generative power. In the construction of the SLC-3DMM, the scans of the Binghamton University 3D facial expression dataset (BU-3DFE) were used. One interesting aspect of this dataset is that it includes sufficient variability in terms of gender, ethnicity, and age, also providing neutral and expressive scans. In particular, expressive scans are given for the six prototypical expressions (i.e., angry, disgust, fear, happy, sad, and surprise), with four different levels of intensity, from small to exaggerated, that also include topological variations, like for open/closed mouth. In order to compute the average model it is necessary that such training scans are posed in dense semantic correspondence. This means that all the scans should have the same number of points, and corresponding points should have the same semantic meaning. For the SLC-3DMM this has been obtained by exploiting the solution first proposed in [5,30]. The idea is to start from the facial landmarks that are annotated for each scan of the BU-3DFE (i.e., 83 landmarks per scan). Such landmarks provide initial points of correspondence across all the scans that are then used to partition the face surface in a set of non-overlapping regions. Each region is re-sampled internally so that the same regions for different scans have the same number of sampling points. Overall this results in scans that have been re-sampled according to a common semantic.

Relying on such set of scans in dense semantic correspondence, the geometry of a generic 3D face in the training set is represented as a vector $f_{i}={\left[x_{1},y_{1},z_{1},\cdots ,x_{m},y_{m},z_{m}\right]}^{T}\in R^{3m}$ that contains the linearized (x,y,z) coordinates of the *m* vertices. Let $F=\left[f_{1}\left|,\cdots ,\right|f_{N}\right]\in R^{3m\times N}$ be the matrix of the *N* training scans, each with *m* vertices arranged column-wise. Then, the difference between each training scan and the average 3D face is computed as

(1) $$m=\frac{1}{N}\sum_{i=1}^{N}f_{i}\;,\;\;\;\;\;v_{i}=f_{i}-m\;,\;\forall \;f_{i}\in F\;.$$

Each $v_{i}$ represents the set of directions that transform the average model m into a training model $f_{i}$. Such $v_{i}$ form the training matrix $V=\left[v_{1}\left|,\cdots ,\right|v_{N}\right]\in R^{3m\times N}$.

The peculiar characteristic of the SLC-3DMM is that it changes the way we look at the training data. Instead of using each $v_{i}$ as a separate training sample, as in the “standard” 3DMM approach, each vertex coordinate is treated independently, that is, the displacements of each coordinate across the *N* scans are used as training samples. So, each sample becomes an *N*-dimensional data point $v_{i}^{\prime }\in R^{N}$ representing the statistics of variation each vertex coordinate is subject to, for a total of 3m training samples. Practically, this is obtained by simply transposing the training matrix V. The estimation of the primary directions and expansion coefficients is formulated as a sparse-coding problem, in which the goal is to find a set of directions that can be sparsely combined to reconstruct the training data. This procedure is summarized in the following.

Let $V^{\prime }=V^{T}\in R^{N\times 3m}$ be the transposed training matrix. We wish to find a set of *k* (k≪3m) primary directions $D\in R^{N\times k}$ and sparse expansion coefficients $C=\left[c_{1}\left|,\cdots ,\right|c_{3m}\right]\in R^{k\times 3m}$ that allow optimally reconstructing the input data, i.e., such that ${∥V^{\prime }-DC∥}_{2}^{2}$ is minimized and C is sparse. To obtain realistic deformations, the coefficients should also be smooth enough to prevent discontinuities. The problem is formulated as

(2) $$\min_{c_{i},D}\frac{1}{3m}\sum_{i=1}^{3m}{∥v_{i}^{\prime }-Dc_{i}∥}_{2}^{2}+\lambda_{1}{∥c_{i}∥}_{1}+\lambda_{2}{∥c_{i}∥}_{2}^{2}\;,\;\;s.t.\;D\geq 0\;,\;C\geq 0\;.$$

This formulation is known as Elastic-net, and it has some properties that make it particularly suitable for this task. In particular, the $\ell_{2}$ regularization encourages the grouping effect [31], that is when the coefficients of a regression method associated with highly correlated variables tend to be equal. This correlation is in terms of displacement direction, and it is caused by the local consistency of motion induced by facial muscles. Together, these two characteristics result in deformations that are both sparse and spatially localized. Finally, it is worth noting that in addition to learn the vertex motions, a positivity constraint is also forced in (2), which induces additional sparsity to the solution by promoting the complementarity of each learned atom [32].

The sparse components C, the average model m, and the weight vector μ constitute the Sparse and Locally-Coherent (SLC)-3DMM. For a more detailed description on the SLC-3DMM construction procedure, the reader can refer to [3].

### 3.2. Fitting the SLC-3DMM to Target Scans

Fitting a 3DMM to a 3D face scan allows a coarse 3D reconstruction of the face, which is obtained by a different parameterization established by the vertex association between the 3DMM and the target. The process is started by a preliminary ICP alignment. Then, the following steps are iterated until a stopping condition is reached (more details can be found in [3]):

1. A vertex correspondence is established between vertices in the 3DMM and the target.
2. A transformation accounting for rotation, scale, and translation is estimated between the average 3DMM S and the target ${\hat{t}}^{c}$:
  (3) $$S={\hat{t}}^{c}\cdot P+T\;,$$
  where $T\in R^{3}$ is the 3D translation, and $P\in R^{3\times 3}$ contains the 3D rotation and scale parameters. P is found in closed-form solving the following least-squares problem:
  (4) $$\underset{P}{\arg\min}{∥S-{\hat{t}}^{c}\cdot P∥}_{2}^{2}\;.$$
  A solution to (4) is given by $P=S\cdot {\hat{t}}^{c†}$, where ${\hat{t}}^{c†}$ is the pseudo-inverse of ${\hat{t}}^{c}$. The translation is then recovered as $T=S-P\cdot {\hat{t}}^{c}$. Rotation and scale matrices $\left[R,S_{c}\right]\in R^{3\times 3}$ can be retrieved applying QR decomposition to P. Using $\left[R,S_{c}\right]$ and T, we re-align both ${\hat{t}}^{c}$ and $\hat{t}$ to S prior to performing the deformation:
  (5) $${\hat{t}}^{c}=\left({\hat{t}}^{c}\cdot R\right)\cdot S_{c}+T\;.$$
3. To deform S, we need to find the optimal set of deformation coefficients $\alpha \in R^{k}$ so that the per-vertex distance between the two point sets is minimized. Similar to other works using a morphable model [5,33,34], we formulate the problem as a regularized least-squares:
  (6) $$\min_{\alpha }{∥{\hat{t}}^{c}-S-C\alpha ∥}_{2}^{2}+\lambda {∥\alpha \circ \mu^{-1}∥}_{2}\;,$$
  where λ is the regularization parameter that allows balancing between the fitting accuracy and smoothness of the deformation. Here we regularize the deformation using the inverse $\mu^{-1}$ so that the contribution of each component is weighed with respect to its average intensity. By pre-computing $X={\hat{t}}^{c}-S$, the solution is found in closed form:
  (7) $$\alpha ={\left(C^{T}C+\lambda \cdot \mathrm{diag}\left({\hat{\mu }}^{-1}\right)\right)}^{-1}C^{T}X\;.$$
  In the equation above, the symbol $\mathrm{diag}({\hat{\mu }}^{-1})$ is used to denote a diagonal matrix with the vector ${\hat{\mu }}^{-1}$ on its main diagonal. S is then deformed applying $m+\sum_{i=1}^{k}c_{i}\alpha_{i}.$ Finally, we estimate the per-vertex error of the deformed model as the average Euclidean distance between each vertex of S and its nearest-neighbor in $\hat{t}$.

The procedure reported above is performed in an iterative way. The stopping criteria are reached when the error between two subsequent iterations falls under a predefined threshold $\tau_{e}$, or after a maximum number of iterations. When the stopping condition is reached, the 3DMM is fit to the target shape. A more detailed description of the fitting procedure is reported in [3].

**Figure 1:**
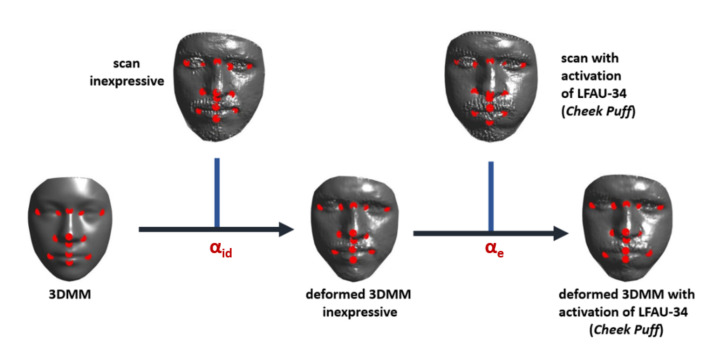
Workflow of the proposed procedure to extract the AU-specific deformation coefficients from the 3DMM fitting. First, the average model is fit on the neutral scan of each subject, retrieving the deformation coefficients $\alpha_{id}$ related to the identity. Then, the neutral model is used in place of the average one to fit scans with AUs activated. The resulting set of deformation coefficients $\alpha_{e}$ encodes the motion related to a particular AU, independently from the identity.

Figure 1 illustrates a fitting example, where the SLC-3DMM has been fit to a target scan using the procedure discussed above. It is possible to observe the SLC-3DMM fitting is quite accurate.

## 4. Learning AU-Specific 3DMM Coefficients

The proposed method to extract the deformation coefficients related to the activation of single AUs is rather simple yet effective. Considering a raw target face t, the deformation coefficients α resulting from the fitting process expounded in Section 3.2 encode both global shape variations (i.e., the identity) along with other motions (i.e., action units activation). In order to extract only the information related to the activation of the AUs, we decouple the two by defining a two-step process. First, the average model m is deformed with the procedure expounded in Section 3.2 to fit the scan in neutral expression $t_{n}$ of an individual, so as to model morphological traits related to the identity and retrieve the related coefficients $\alpha_{id}$. The fitted 3DMM results in a neutral model $s_{n}$ that has the same topology of m. However, it still represents an approximation of the target face. In order to ameliorate it and improve the subsequent steps, we map each vertex of $s_{n}$ to its nearest-neighbor vertex in t. In doing so, we obtain a neutral model ${\hat{s}}_{n}$ that still has the same topology of m, but each point is sampled from t, thus representing the actual target surface. Then, ${\hat{s}}_{n}$ is used in place of m to fit raw scans $t_{e}$ of the same subject with action units activation. In order to reduce the impact of possible misalignment that could impair the estimation of the coefficients, we first align the nose-tips of ${\hat{s}}_{n}$ and $t_{e}$, and perform a rigid ICP to account for slight rigid roto-translations. Then, we again apply the fitting of Section 3.2. In this way, we obtain a set of coefficients $\alpha_{e}$ that encode the deformation associated with a particular AU. The process is illustrated in Figure 1.

Differently from [4] where the process is guided by a sparse set of landmarks, thus limiting the possible deformations that can be captured, here the fitting is iterative and optimizes the shape with respect to the whole point-cloud. Hence, for each non-neutral scan we obtain a variable number $N_{i}$ of coefficients $\alpha_{e}$, which depends on the iterations. Optimizing with respect to the point-cloud has the advantage of capturing finer deformations of the face surface; on the other hand, minor deformations due to slight misalignment or sensor-noise could be captured while estimating the coefficients $\alpha_{e}$. To remove such information from the coefficients, we experimented with a few solutions, which include (i) Taking the maximum of the coefficients across the iterations, (ii) setting a threshold to remove the effect of minor deformations, (iii) taking the coefficients of the first iteration only, and (iv) averaging the coefficients across the iterations. The latter solution resulted in the best performance. This is because such smaller deformations account for very small vertex displacements, and they can be well approximated by a zero-mean Gaussian noise. So, by taking the average we cancel their effect to a great extent. The magnitude of the deformation needed to compensate slight shape differences are also relatively smaller than those associated with the AU modeling. Ultimately, in this way we are able to associate a single vector of coefficient to each sample.

For each subject, we now have a set of coefficients $\alpha_{e}$ with the corresponding AU label. We use these to train a Support Vector Machine (SVM) classifier. In particular, we chose the C-Support Vector Classification. To address the multi-class problem, where each of the *C* classes is one AU, we employ the standard One-vs-Rest strategy, which splits a multi-class problem into one binary classification for each class, for a total of *C* classifiers. We train the SVM with the RBF kernel, which is in the form of a Gaussian function, defined as

(8) $$K_{RBF}\left(x,x^{\prime }\right)=\exp\left(-\frac{{∥x-x^{\prime }∥}^{2}}{2\sigma^{2}}\right)\;,$$

where σ is a free parameter, and *x* and $x^{\prime }$ represent a feature vectors in some input space, i.e., α vectors in our case.

## 5. Experimental Results

We conducted experiments using the Bosphorus database, that includes 105 subjects performing 24 facial AUs activation (not every subject has all the 24 action units), for a total of 1879 scans. All the 24 AUs were employed for training and testing the SVM classifier. For training and testing, we used the Leave-One-subject-Out Cross Validation, or LOOCV. In particular, the scans of each subject are used once as a test set (Singleton), while the remaining subjects form the training set. The final results are the average over all the test subjects. Features were normalized to unitary L2-norm and standardized by removing the mean and scaling to unit variance prior to training the SVM. The centering and the scaling were done independently on each feature by computing the relevant statistics on the samples in the training set. Features were further processed by applying a dimensionality reduction based on PCA, retaining a number of dimensions carrying the 95% of the variance.

In the experiments, we compared the AU detection accuracy obtained as a result of performing the fitting with the SLC-3DMM described in Section 3 and the standard PCA-3DMM. We chose the configurations that were reported to perform best in [3], which are SLC with 50 components (also named SLC-50), SLC with 300 components (SLC-300), and PCA with 50 components (PCA-50). After that, we also tested whether the two could bring complementary information, and we performed an early fusion of the $\alpha_{e}$ coefficients among SLC-50 with PCA-50 (SLC-PCA-50) and SLC-50 with SLC-300 (SLC-50-300). The fusion was performed by concatenating the vectors from the two modalities. Following the standard convention, results are reported in terms of confusion matrices, F1-score, accuracy, and Area Under the Receiver Operating Characteristic (ROC) Curve (AUC). The former are widely used in facial expression analyses to understand the distribution of the classifier predictions and the properties of the features. F1-score, instead, represents the harmonic mean of precision and recall, while the AUC provides an aggregate measure of a classifier performance for all possible decision thresholds.

Previous works in the literature reported results on Bosphorus only in terms of AUC. So, in the following, we first report a comparison between the PCA-50 and SLC-50 methods in terms of accuracy and F1-score, and we discuss the relevant differences. Then, we provide a more comprehensive evaluation of the general performance of the proposed framework in comparison with the literature using the AUC measure.

### 5.1. Comparing SLC and PCA Deformation Components

**Figure 2:**
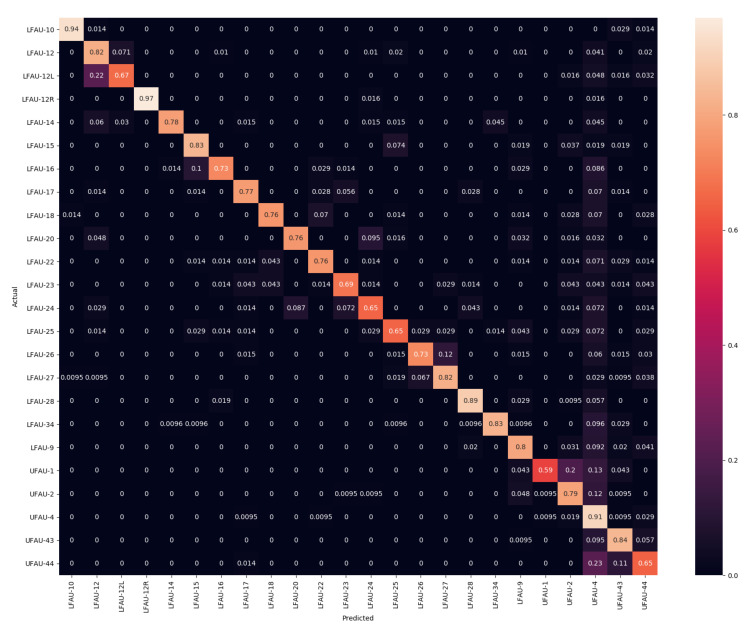
Confusion Matrix using PCA with 50 components.

**Figure 3:**
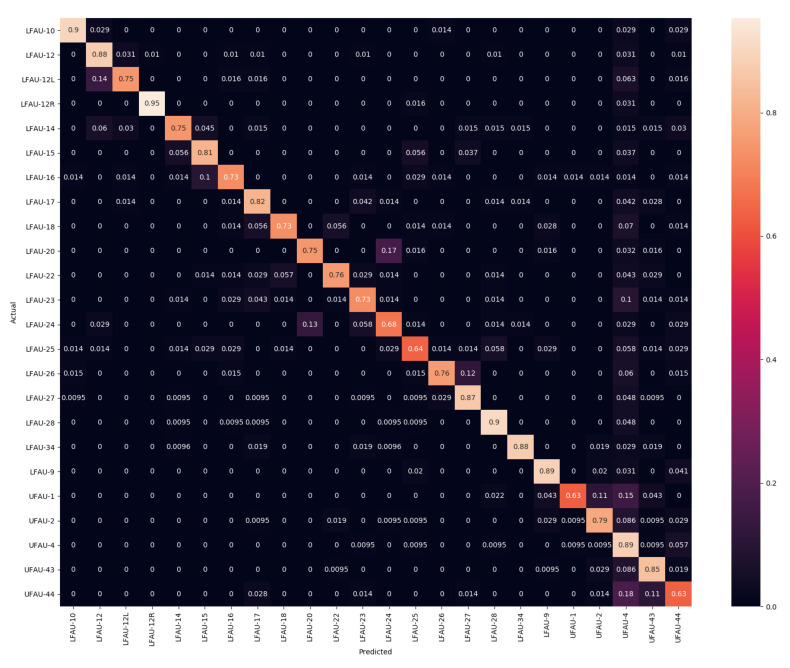
Confusion Matrix using SLC with 50 components.

**Figure 4:**
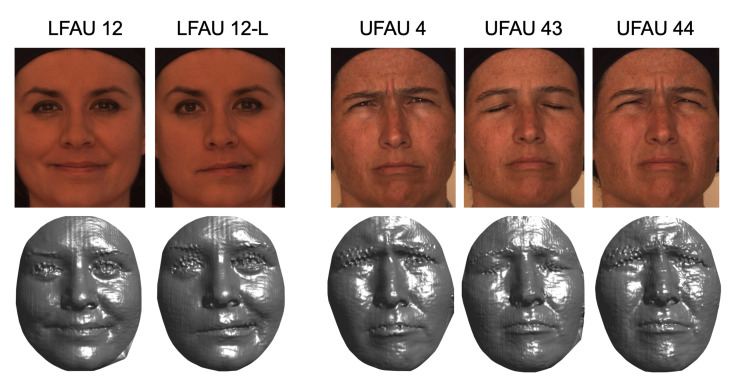
Problematic AUs: LFAU12 L (left) is the asymmetric version of LFAU12, which is complex to capture. UFAU4, UFAU44, and UFAU43 are very similar and difficult to distinguish in 3D, whereas the RGB texture can help to disambiguate by providing additional information on the eyes region.

In this section, we evaluate the differences between the SLC and PCA components, which significantly differ in terms of type of deformation; SLC applies sparse and localized deformation, while PCA has global support on the face, and each component moves all the vertices of the 3DMM. Confusion matrices for PCA-50 and SLC-50 are reported in Figure 2 and Figure 3, respectively. Each row of the confusion matrix represents instances in the actual class, while each column represents instances of the predicted class. Therefore, a more accurate classification is represented by a confusion matrix with the diagonal line that has the higher percentages, but it can provide useful information on the behavior of the classifier. For example, tests indicate that LFAU-12L gets a little confused with the LFAU-12, which is plausible as both concern the lip corner puller, the first one being the asymmetric left lip corner. It turns out in this case that the SLC components, thanks to their sparse nature, better capture such slight difference and provide more accurate detection. The same occurs for UFAU-1, or Inner Brow Raiser, and UFAU-2, or Outer Brow Raiser, which get less confused when using the SLC components. We finally note a generally lower accuracy for the UFAU-44, or Squint. For this particular action unit, we find the absence of surface details in the eyes region of the 3DMM a potential source of error in this case. Examples of these are shown in Figure 4. In particular, the reader can appreciate the very slight differences occurring between the set of action units involving the eyebrows, which is most evident for the sole 3D shape.

A similar behavior comes out from the results in Table 1, where F1 and accuracy scores are reported. They show an excellent level for the most of the action units. Again, the worst cases are related to LFAU-12L, UFAU-1, and UFAU-44 in all the cases, which get confused with other very similar action units, as evidenced by the confusion matrices. SLC components perform generally slightly better than PCA ones, mostly for those AUs that involve the movement of smaller face areas. This represents a piece of evidence that modeling facial deformations locally can be beneficial. Indeed, we observe the largest accuracy increase being related to such action units (e.g., LFAU-12L for which there is an accuracy improvement of 8%). Overall, the proposed framework to detect the activation of action units in 3D faces provides accurate results. There are yet some problematic action units that are more difficult to detect. However, we will see in the next section that the approach is rather robust and provides a good recall on all the action units compared to other previous methods. A noticeable aspect of this solution is that these outcomes are a direct result of the fitting process and so derive from the analysis of the vertices’ motion, eliminating the need to design, choose, and compute any surface descriptors.

The Bosphorus database also includes some annotations of multiple action units activation and prototypical expressions, which can be viewed as the result of the activation of multiple action units. We performed an additional experiment on these samples aimed at verifying the behavior of the proposed method in case of more complex facial deformations. To this aim, we applied the method described in Section 4 to recover the deformation coefficients. Just the same as for the AUs, we cast this learning problem as a single-label multi-class problem; thus, we trained the SVM to classify either the one of the six expressions, i.e., Anger, Disgust, Fear, Happy, Surprise, Sadness, or the two available AU combinations. Recognizing the expression in terms of a single action unit would have required to change the learning problem into a multi-label, multi-class, which was not the scope of this work. Results are reported in Table 2 and show macro-expressions are fairly accurately recognized, again with the SLC solution performing slightly better in most of the cases. It is interesting to observe that the expression where the most significant improvement occurred was “disgust”, which is heavily related to the activation of AU9, that is “nose wrinkler”. Incidentally, that is one AU where a larger gap between PCA and SLC occurred (see Table 1). The opposite happened for “Anger”, which is instead related to AU4 (eyebrow lowerer) and partially to AU44 (squint).

### 5.2. Comparison with the State-of-the-Art

**Figure 5:**
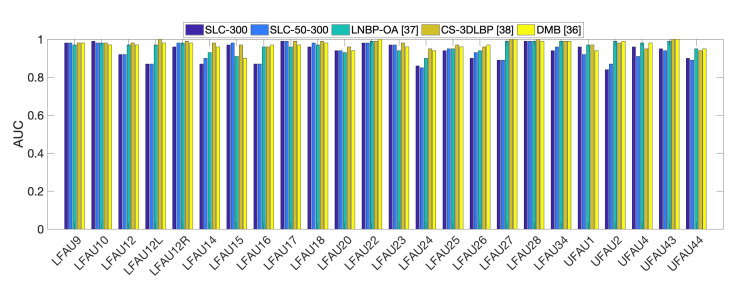
ROC-AUC scores comparing our approach with SLC-3DMM against the state-of-the-art.

In this section, we report a comparison with state-of-the-art methods on the Bosphorus database. All the previous works report results in terms of Area Under the Receiver Operating Characteristic Curve (ROC-AUC), which is a fundamental tool for diagnostic accuracy evaluation, and so we compared in terms of this measure. We computed and reported AUC values for each action unit separately. Results for the compared approaches have been collected from the original papers and are reported in Table 3 and Figure 5. It is relevant here to comment about a significant difference between the proposed approach and the compared works. The 3DMM that is used to fit the raw scans of the Bosphorus and retrieve AU-specific deformation coefficients as described in Section 4 was built from the fully registered scans of a different database, that is the BU-3DFE [35]. So, despite the SVM classifier being trained on Bosphorus training splits, we argue that, in a way, this represents a challenging cross-dataset setting. This because the relevant statistics of face variations captured by the deformation components are learned on a different set of 3D faces; these are carried by the 3DMM and transferred to the Bosphorus faces so to encode the deformations. Note that the BU-3DFE database contains completely different subjects, only prototypical expressions, and does not include single action units activation. In the compared approaches, all the surface descriptors, e.g., 3DLBP, Cs-3DLB, that are used to train the classifiers are directly computed on the Bosphorus scans. We argue this can impair the generalization ability of the classification inasmuch as scans collected from different devices, i.e., from different datasets, present very different surface characteristics in terms of resolution, noise, and topology. Unfortunately, no previous works report cross-database results on Bosphorus; however, in [36], a cross-database experiment was conducted by training the classifiers with descriptors computed on Bosphorus and testing on a different database (D3DFACS). In this case, AUC measures dropped around 10%, demonstrating the difficulty of generalizing to shapes from different databases, which the authors in [36] ascribe to the diverse levels of mesh smoothness that inevitably affect the generalization performance. Our solution, instead, relies on deformation coefficients that encode 3D motion of points.

In Table 3, we also report results obtained with SLC-300 and the two early fusion solutions. The general trend is similar to that of the previous measurements, both in the best and worst cases. Results show our method performed competitively with respect to state-of-the-art approaches. The best performance was obtained using SLC-300 and the early fusion between SLC-50 and SLC-300. As reported in [3], increasing the number of SLC-components induces an indirect effect on the deformation extent. Practically, when more components are used, the area of the deformation is reduced. The higher general performance can be ascribed to this fact, being the activation of facial action units related to the movement of localized face regions.

Figure 5 reports also a graphical representation of the results (only best configurations as from Table 3 are reported for clarity of visualization). It turns out evidently that some AUs were more critical, e.g., LFAU-12L, LFAU-14, LFAU-16, while some others were very accurately recognized, e.g., LFAU-10, LFAU28, LFAU-17. We argue this is likely due to the difficulty of reproducing some particular movements when performing the 3DMM fitting. These include asymmetric motions (LFAU-12L), or subtle and peculiar movements like dimpler (LFAU-14) or lip presser (LFAU-24). A different aspect that could represent a concurrent cause of this behavior is the possible bias induced by the characteristics of the BU-3DFE scans, from which our 3DMM is built. As discussed previously, the BU-3DFE contains only prototypical expressions. In this regard, it is possible that some action units that are less correlated to facial expressions are being recognized with more difficulty. This suggests that performance could be improved using a more descriptive 3DMM. Indeed, those AU that instead are more related to expressions, get very good detection results, even superior to the compared approaches. Finally, the strong level of noise of many Bosphorus scans, as for example those subjects with the beard, further complicated the problem.

### 5.3. Generating Action Units Activation

**Figure 6:**
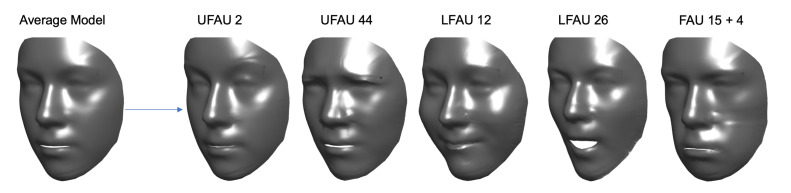
Examples of synthetically generated AUs. The average model m is deformed with AU-specific $\alpha_{e}^{AU}$ coefficients. UFAU 2—Outer brow raiser, UFAU 44—Squint, LFAU 12—Lip corner puller, LFAU 26—Jaw drop. Rightmost: combination of UFAU 4 (eyebrow lowerer) and LFAU 15 (lip corner depressor).

In this section we show that by applying the process described in Section 4, the estimated deformation coefficients $\alpha_{e}$ effectively encode the information related to the activation of specific action units, and they can be used to generate 3D faces with synthetic action units activation. For each action unit, we accumulated all the related coefficients across all the *M* subjects, and we estimated a set of AU-specific coefficients as $\alpha_{e}^{AU}=\frac{1}{M}\sum_{i=1}^{M}\alpha_{e}^{i}$. We used these coefficients to deform the average model as $m+\sum_{i=1}^{k}c_{i}\alpha_{i}^{AU}$, and we generated new shapes with a specific action unit. Some examples are shown in Figure 6. Each set of coefficients $\alpha_{e}^{AU}$ accurately reproduced the motion induced by different action units, resulting in realistic and smooth 3D faces. In addition, note that regions of the face that were not involved in the motion were deformed to a very small extent. For example, reproducing the UFAU 2, which corresponds to raising the outer eyebrows, just slightly modified the mouth region, but it left the structural traits of the face unchanged, e.g., nose shape.

Event though the SLC components are meant to apply localized deformations, when performing the fitting as expounded in Section 3.2, all the 3DMM vertices were involved in the deformation; thus, it is likely that some residual information resulting from the fitting leads to smaller deformations being performed overall the face. It is evident from Figure 6 that these are yet very slight. The complementary nature of action units also allows us to fuse coefficients $\alpha_{e}^{AU}$ of different action units so to generate combined samples. Obviously, it would be pointless to combine action units involving the same face region, e.g., UFAU 2 and UFAU 4, which both control the eyebrows motion but in opposite directions. The rightmost example of Figure 6 corresponds to a face generated by combining the coefficients of UFAU 4 (eyebrow lowerer) and LFAU 15 (lip corner depressor). To obtain it, we simply computed the average of the two without any further processing. This is evidence that the proposed two-step fitting process allows us to extrapolate AU-specific deformation coefficients, disentangling the identity and expressions components.

**Figure 7:**
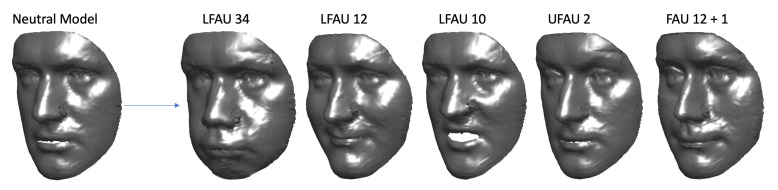
Examples of synthetically generated AUs. The neutral model ${\hat{s}}_{n}$ of a subject is deformed with AU-specific $\alpha_{e}^{AU}$ coefficients. LFAU 34—Cheek Puff, LFAU 12—Lip corner puller, LFAU 10—Upper Lip Raiser, UFAU 2—Outer Brow Raiser. Rightmost: combination of UFAU 1 (inner brow raiser) and LFAU 12 (Lip corner puller).

**Figure 8:**
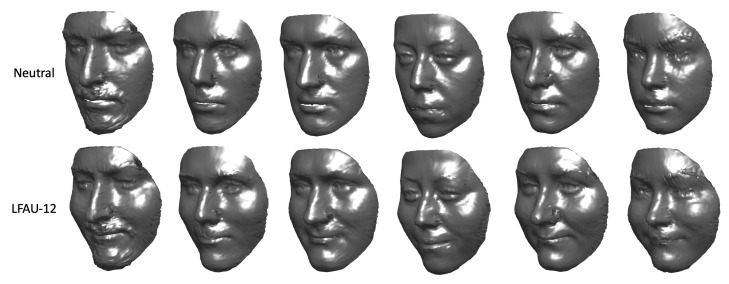
Examples of synthetically generated AUs. The neutral scan of different subjects is deformed with the LFAU-12 AU-specific $\alpha_{e}^{AU}$ coefficients.

To further demonstrate the retrieved coefficients effectively encode particular action units deformation and are independent of the identity, we applied some AU-specific coefficients $\alpha_{e}^{AU}$ as described above, to neutral models ${\hat{s}}_{n}$ instead of the average model m. In Figure 7 an example is shown for one subject. Similarly to Figure 6, the reader can appreciate the fact the neutral model ${\hat{s}}_{n}$ is deformed in a way that the action units are activated, but still the identity does not change. To explicitly show this is not simply a particular case but instead applies to arbitrary subjects, we applied the $\alpha_{e}^{AU}$ related to a specific AU (LFAU-12) to different identities. Figure 8 shows that, irrespective of the subject, we obtained a natural deformation, while maintaining stable morphological traits.

**Figure 9:**
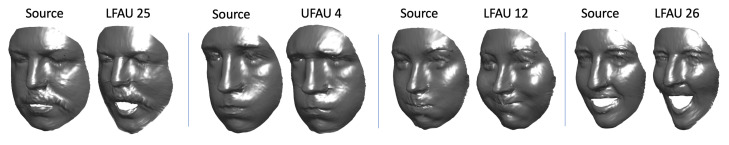
Examples of synthetically generated AUs on scans from FRGCv2.0. The neutral scan of different subjects is deformed with AU-specific $\alpha_{e}^{AU}$ coefficients: LFAU-25—lips part, UFAU 4—eyebrow lowerer, LFAU 12—lip corner puller, LFAU 26—jaw drop.

Finally, in order to showcase the versatility of the proposed approach and the high generalization of the learned coefficients, we applied the fitting approach described in Section 3.2 to some raw scans of the FRGCv2.0 dataset [39] and obtained the corresponding neutral models ${\hat{s}}_{n}$. Then, we used the $\alpha_{e}^{AU}$ coefficients described above obtained from the Bosphorus scans to apply AU-deformations to the FRGC ones. Figure 9 shows that even changing the target dataset, the applied deformations were realistic and well maintained the identity of the subject. Furthermore, from the examples in Figure 9, we observe that the neutral models ${\hat{s}}_{n}$ actually did not necessarily need to be in neutral expression. For example, the second and third columns show neutral models with puffed up cheeks, while the scan in the rightmost column is smiling; this fact does not prevent to apply additional facial movements and generate even more complex shapes.

## 6. Discussion

The proposed framework for AU detection on 3D faces showed both advantages and limitations. As observed in Section 5.1, the information carried out by the 3DMM deformation coefficients cannot completely capture very slight differences occurring in the activation of AUs involving highly overlapping face regions. We argue that the surface noise as induced by scanners represents a concurrent cause of this behavior. The sparse SLC formulation was more effective than the standard PCA in these cases, being specifically designed to apply localized deformations. On the other hand, we reported results that are comparable with that of approaches relying on multiple features extracted from the face surface. We find being independent from any surface descriptor represents a valuable advantage of our solution. This because different capturing devices result in 3D faces that are highly variable in terms of, for example, number and disposition of vertices, surface granularity, noise, and general topology. Even though descriptors such as 3DLBP and variants are usually computed after converting the shape into a 2D depth representation, the above differences could still negatively impact the computation of the descriptors, as also discussed in [36]. Finally, we showed that, other than being able to precisely classify the different AUs, we jointly learn AU-specific deformation coefficients by decoupling identity and expression from the 3D faces. This property allows us to control the learned coefficients and generate new faces with arbitrary AUs. This opens a lot of possible applications, from data augmentation to animation.

Another evident limitation of the current learning approach is that at least one sample in the neutral expression of each subject is required to perform the first step of the fitting process, so to account for the identity component. On the other hand, we showed in Section 5.3 that AU-specific representative sets of coefficients are easily computed from the available samples and are used to replicate AU deformations. The same process could be applied to the identity part, i.e., $\alpha_{id}$, so to retrieve another representative set of coefficients, this time modeling the general identity component. It would be then possible, given a non-neutral target scan, to perform the fitting and separate the identity and expression components a posteriori, provided the two representative sets. As we did not address this issue in the current work, we aim at further developing the method to remove the two-step constraint.

## 7. Conclusions

In this manuscript, a method for detecting and classifying the activation of facial action units (AU) on 3D faces was presented. The detection is achieved by isolating the AU-specific deformation coefficients of a 3D Morphable Face Model (3DMM) by means of a two-step fitting process. By first adapting the 3DMM to the neutral scan of an individual we are able to remove the identity component and subsequently identify a set of AU-specific deformation coefficients. We showed that these coefficients can be used to train a simple SVM classifier to detect the activation of 24 different AUs. We further showed that, in this particular scenario, modeling facial deformation locally using a sparse variant of the 3DMM formulation is beneficial. The reported results show that competitive performances with respect to state-of-the-art solutions are achieved, though experimenting in a challenging cross-dataset setting, and without computing any surface descriptors.

## Figures and Tables

**Table 1:** F1 and accuracy scores across all the AUs, for the PCA-50 and SLC-50 models.

	F1-Score		Accuracy	
**AUs**	**PCA-50**	**SLC-50**	**PCA-50**	**SLC-50**
LFAU-10	**0.97**	0.95	**0.94**	0.90
LFAU-12	0.90	**0.93**	0.82	**0.88**
LFAU-12L	0.80	**0.85**	0.67	**0.75**
LFAU-12R	0.98	0.98	**0.97**	0.95
LFAU-14	**0.87**	0.85	**0.78**	0.75
LFAU-15	**0.91**	0.90	**0.83**	0.81
LFAU-16	0.84	0.84	0.73	0.73
LFAU-17	0.87	**0.90**	0.77	**0.82**
LFAU-18	**0.86**	0.85	**0.76**	0.73
LFAU-20	**0.86**	0.85	**0.76**	0.75
LFAU-22	0.86	0.86	0.76	0.76
LFAU-23	0.81	**0.84**	0.69	**0.73**
**AUs**	**PCA-50**	**SLC-50**	**PCA-50**	**SLC-50**
LFAU-24	0.79	**0.81**	0.65	**0.68**
LFAU-25	**0.79**	0.78	**0.65**	0.64
LFAU-26	0.84	**0.86**	0.73	**0.76**
LFAU-27	0.90	**0.93**	0.82	**0.87**
LFAU-28	0.94	**0.95**	0.89	**0.90**
LFAU-34	0.91	**0.93**	0.83	**0.88**
LFAU-9	0.89	**0.94**	0.80	**0.89**
UFAU-1	0.74	**0.77**	0.59	**0.63**
UFAU-2	0.88	0.88	0.79	0.79
UFAU-4	**0.96**	0.94	**0.91**	0.89
UFAU-43	0.91	**0.92**	0.84	**0.85**
UFAU-44	**0.79**	0.78	**0.65**	0.63
μ	0.87	**0.88**	0.78	**0.79**

**Table 2:** F1 and accuracy scores for combined AUs and standard expressions.

	F1-Score		Accuracy	
**AUs**	**PCA-50**	**SLC-50**	**PCA-50**	**SLC-50**
AU26-AU12L	0.96	0.96	0.92	0.92
AU22-AU25	1.00	1.00	1.00	1.00
**Expression**	**PCA-50**	**SLC-50**	**PCA-50**	**SLC-50**
Anger	**0.96**	0.94	**0.93**	0.88
Disgust	0.81	**0.88**	0.69	**0.78**
Fear	0.84	**0.85**	0.73	**0.74**
Happy	0.93	**0.95**	0.86	**0.91**
Surprise	0.88	0.88	0.79	0.79
Sadness	0.94	**0.95**	0.88	**0.89**

**Table 3:** ROC-AUC for each action unit, the different models (PCA-50, SLC-50, SLC-300), and the fusion of their coefficients (SLC-PCA 50, SLC 50–300). Results are compared with respect to the state-of-the-art.

AUs	PCA 50	SLC 50	SLC 300	SLC-PCA 50	SLC 50–300	3DLBP [37]	3DLBP + LNBP_OA_ [37]	3DLBP + LNBP_TA_	CS-3DLBP + RbG [38]	DMB [36]
LFAU-10	0.90	0.96	0.99	0.90	0.98	0.98	0.98	0.98	0.98	0.97
LFAU-12	0.85	0.85	0.92	0.85	0.92	0.96	0.97	0.96	0.98	0.97
LFAU-12L	0.72	0.90	0.87	0.72	0.87	0.96	0.97	0.97	1.00	0.98
LFAU-12R	0.94	0.99	0.96	0.95	0.98	0.98	0.98	0.97	0.99	0.98
LFAU-14	0.87	0.88	0.87	0.88	0.90	0.91	0.93	0.91	0.98	0.96
LFAU-15	0.93	0.94	0.97	0.93	0.98	0.85	0.91	0.84	0.97	0.90
LFAU-16	0.83	0.88	0.87	0.84	0.87	0.97	0.96	0.97	0.96	0.97
LFAU-17	0.94	0.86	0.99	0.94	0.99	0.93	0.96	0.95	0.99	0.97
LFAU-18	0.96	0.95	0.96	0.96	0.98	0.97	0.97	0.97	0.99	0.98
LFAU-20	0.89	0.94	0.94	0.89	0.94	0.90	0.93	0.93	0.96	0.94
LFAU-22	0.90	0.92	0.98	0.92	0.98	0.99	0.99	0.99	0.99	1.00
LFAU-23	0.88	0.89	0.97	0.88	0.97	0.95	0.94	0.95	0.98	0.96
LFAU-24	0.93	0.84	0.86	0.94	0.85	0.89	0.90	0.90	0.95	0.94
LFAU-25	0.89	0.87	0.94	0.89	0.95	0.93	0.95	0.95	0.97	0.96
LFAU-26	0.93	0.92	0.90	0.93	0.93	0.93	0.94	0.95	0.96	0.97
LFAU-27	0.86	0.95	0.89	0.86	0.89	0.99	0.99	0.99	1.00	1.00
LFAU-28	0.97	0.90	0.99	0.97	0.99	0.98	0.99	0.98	1.00	0.99
LFAU-34	0.98	0.97	0.94	0.99	0.96	0.99	0.99	0.99	0.99	0.99
LFAU-9	0.97	0.96	0.98	0.97	0.98	0.98	0.97	0.98	0.98	0.98
UFAU-1	0.96	0.89	0.96	0.96	0.92	0.93	0.97	0.91	0.97	0.94
UFAU-2	0.86	0.90	0.84	0.87	0.87	0.98	0.99	0.98	0.98	0.99
UFAU-4	0.80	0.79	0.96	0.80	0.91	0.96	0.98	0.97	0.95	0.98
UFAU-43	0.94	0.99	0.95	0.94	0.94	0.99	0.99	1.00	1.00	1.00
UFAU-44	0.80	0.75	0.90	0.80	0.89	0.95	0.95	0.94	0.94	0.95
μ	0.90	0.90	0.93	0.90	**0.94**	0.95	0.96	0.96	**0.98**	0.97

## Data Availability

Not applicable.
